# Proteomic Analysis of Human Follicular Fluid Reveals the Pharmacological Mechanisms of the Chinese Patent Drug Kunling Pill for Improving Diminished Ovarian Reserve

**DOI:** 10.1155/2022/5929694

**Published:** 2022-05-28

**Authors:** Haiyan Wang, Dan Cao, Meixian Wang, Yanbin Shi, Bowen Wei, Shiyuan Jiang, Yangyu Jiang, Hui Lian, Xiaoou Xue, Zhiqiang Ma, Jian Li

**Affiliations:** ^1^Department of Histology and Embryology, School of Traditional Chinese Medicine, Beijing University of Chinese Medicine, Beijing 102488, China; ^2^Reproductive and Genetic Medical Center, Dalian Women and Children's Medical Group, Dalian 116000, Liaoning, China; ^3^Dongzhimen Hospital, Beijing University of Chinese Medicine, Beijing 100700, China; ^4^School of Chinese Pharmacy, Beijing Key Lab for Quality Evaluation of Chinese Materia Medica, Beijing University of Chinese Medicine, Beijing 102488, China

## Abstract

**Objective:**

To explore the pharmacological mechanism of a Chinese patent drug (Kunling Pill (KLP)) on improving diminished ovarian reserve based on proteomic analysis.

**Methods:**

A total of 18 patients divided into three groups (the normal ovary reserve (NOR), diminished ovary reserve (DOR), and KLP groups) undergoing assisted reproductive technology by standard ovarian stimulation protocols were recruited to collect follicular fluid. Data-independent acquisition mass spectrometry was used to identify differentially expressed proteins by nano-LC-MS/MS. Bioinformatic analysis was conducted to predict the functions and pathways of the identified proteins. Clinical, hormonal, and biochemical parameters were also analyzed in the three groups.

**Results:**

A total of 144 differentially expressed proteins were screened out, including 56 proteins that were downregulated and 88 proteins that were upregulated in the DOR group compared with the NOR group, while 27 proteins were shared in the KLP-treated group. Among them, 10 proteins were upregulated and 17 proteins were downregulated in the KLP-treated group compared with the DOR group. The most enriched biological processes accounted for 28 GO terms, including cellular process, biological regulation, metabolic process, and regulation of biological process. Significant pathways were associated with fatty acid elongation, fatty acid degradation, fatty acid metabolism, nicotinate and nicotinamide metabolism, and valine, leucine, and isoleucine degradation.

**Conclusion:**

Our study provides the proteome profiles of human follicular fluid from DOR patients treated by KLP. Functional analyses of proteome datasets revealed that core proteins (SAA1, MIF, and PRDX5) and related pathways (fatty acid metabolism, nicotinate and nicotinamide metabolism, and tyrosine and purine metabolism) are possible pharmacological mechanisms through which KLP improves DOR. Therefore, these findings may help better understand the complex mechanisms through which DOR is treated by the Chinese patent drug KLP.

## 1. Introduction

With lifestyle changes, a trend for postponement of pregnancy due to later marriage, and later childbearing, the number of infertile couples in China has increased. According to clinical reports, more than 20% of women with infertility are diagnosed with diminished ovarian reserve (DOR) in China (ref. in Chinese, not shown) [1, 2]. The main reasons for DOR include genetics, aging, stress, endometriosis, reproductive endocrine disorders, chemotherapy and radiotherapy, excessive drinking, autoimmune diseases, chemical toxins, ovarian induction agents, certain systemic diseases, previous mumps infection, and some unclear etiologies [3]. Although there is no generally accepted definition of DOR, it has been described as a pathophysiological ovarian state characterized by poor fertility outcome, poor ovarian response, and an abnormal ovarian reserve [4, 5].

In the clinic, many treatment approaches have been attempted to improve reduced ovarian reserves, such as estrogen replacement therapy [6, 7], dehydroepiandrosterone [8], resveratrol [9], coenzyme Q10 [10], and stem cell therapy [11]. However, the evidence to support these interventions is weak. Some challenges include unexpected adverse reactions such as breast cancer, heart disease, and thrombus and an increased incidence of estrogen or androgen-related malignancies [12]. Therefore, researchers have sought safer and more effective medicines for treating DOR.

In traditional Chinese medicine (TCM) theory, DOR is categorized as a liver depression and kidney deficiency syndrome (Gan Yu and Shen Xu Zheng), thereby reinforcing the TCM notion that the kidney and liver are important therapeutic targets for DOR. Kunling Pill (KLP, also called Kunling Wan), a standardized Chinese patent drug approved in China since 2004, has been widely used for the treatment of polycystic ovary syndrome (PCOS) [13] and DOR and for improving pregnancy outcome in the clinic (ref. in Chinese). However, little is known regarding its underlying mechanisms in treating DOR. One reason for this is that the formulation of KLP involves complex components (see supplemental data, S1), leading to variability in its ingredients. Another reason is that conventional research methods applied to TCM preparations face many difficulties due to the multiple components and multiple targets of preparation such as KLP.

Proteomics, a core technology in the current postgenomic era, plays an important role in discovering biomarkers, diagnosing disease phenotypes, and revealing the pharmacological mechanisms of various interventions [14, 15]. In recent decades, the technology and instruments for proteomics have made rapid progress, including mass spectrometry (MS) technology, protein fragmentation techniques, and bioinformatics [16, 17]. In this study, a data-independent acquisition mass spectrometry (DIA-MS) technique was used to scan and identify all peptides in the human follicular fluid (FF) in order to explore the possible pharmacological mechanisms of KLP in the treatment of DOR.

The FF is formed in the secondary follicle stage (antral follicles) and provides the microenvironment surrounding the growing oocyte. The FF contains complex materials, such as proteins and cytokines, which can provide unique insights into the processes regulating healthy follicle development [18]. Alterations in the proteomic signature of the FF might therefore reveal the molecular mechanisms involving small antral follicle-associated proteins and oocyte maturation-associated proteins and might also help to decipher the underlying pathophysiology of ovarian disorders [19–21]. Importantly, in the in vitro fertilization (IVF) process, the FF can be easily obtained during the extraction of oocytes from the follicle, which makes it a useful source for experimental research. Proteomic approaches have been used to study the pathological mechanism of PCOS [22–24], endometrial cysts [25, 26], poor ovarian response [27], ovarian hyperstimulation syndrome [28], and recurrent abortion [29], but they have not been applied to DOR. The incidence of DOR has been increasing, and it shows a trend for occurring in younger patients, but it has been difficult to determine the effect of DOR on FF function. This study aimed to identify protein expression changes in the FF of DOR patients, and our results strongly indicate that proteomic expression patterns in the FF undergo significant alterations in DOR patients.

## 2. Materials and Methods

### 2.1. Experimental Design and Workflow

**Figure 1:**
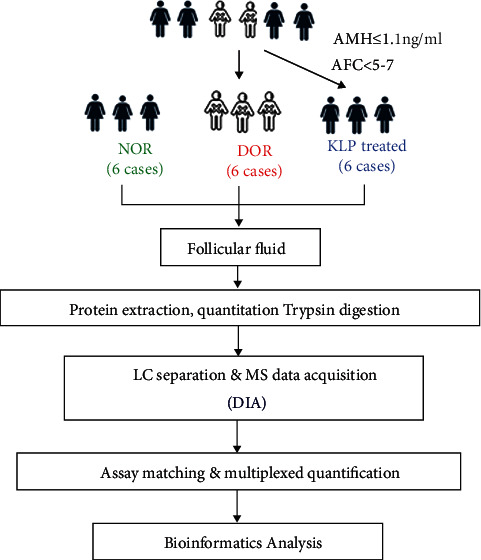
Experimental design and workflow.

The experimental design and workflow are shown in Figure 1. In brief, a total of 18 patients undergoing assisted reproductive technology (ART) by standard ovarian stimulation protocols were recruited to collect FF. Among them, 6 patients had normal ovary reserve (NOR), 6 patients had diminished ovary reserve (DOR), and 6 patients had DOR and were treated with KLP (lot # 20191022) for 3 months.

All patients were recruited from the Reproductive and Genetic Medical Center, Dalian Municipal Women and Children's Medical Center (Group), from March 2020 to March 2021. The inclusion criteria were anti-Müllerian hormone (AMH) ≤ 1.1 ng/ml and/or antral follicle count <5–7 and age <40 years. The exclusion criteria were patients with PCOS, abnormal liver and kidney function, endometriosis, endocrine diseases, or chromosomal abnormalities.

This work was approved by the Ethics Committee of Dalian Municipal Women and Children's Medical Center (No. 2020010), and all participants gave written informed consent.

All participants were infertile women who were scheduled for their first IVF/intracytoplasmic sperm injection cycle. Of these, 6 cases had NOR and were used as the control group, 6 cases had DOR and were used as the disease control, and 6 cases had DOR and were treated with KLP as the experimental group.

### 2.2. Serum Sex Hormone Levels and Embryo Quality Determination

Levels of serum follicle-stimulating hormone (FSH), estradiol (E_2_), and AMH were determined by an automated multianalysis system with a chemiluminescence instrument (DX1800 Beckman Coulter, USA).

**Figure 2:**
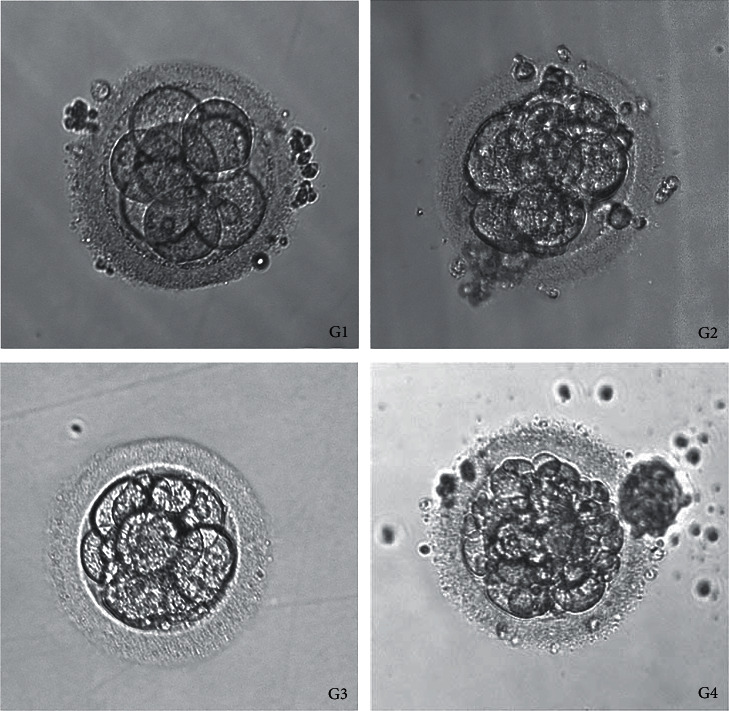
Representative images of embryos of different grades. *Note.* Grade 1 embryo: blastomeres of equal size, regular morphology, bright, and no fragments. Grade 2 embryo: blastomeres of unequal size and/or fragmentation <10%. Grade 3 embryo: 10–50% fragmentation. Grade 4 embryo: fragmentation >50%.

Embryos were divided into four grades based on pronuclear stage score, development speed, number of blastomeres, size, morphology, cytoplasm fragment ratio, and embryo quality score of the cleavage stage. Grade 1 embryo is as follows: blastomeres of equal size, regular morphology, bright, and no fragments. Grade 2 embryo is as follows: blastomeres of unequal size and/or fragmentation <10%. Grade 3 embryo is as follows: 10–50% fragments. Grade 4 embryo is as follows: fragmentation >50%. Embryos with more than six cells at grade 1 or 2 on day 3 after egg collection were defined as high-quality embryos. Embryos with less than 30% fragmentation were available and could be transplanted or frozen (Figure 2).

### 2.3. Collection and Preparation of FF

We used a conventional procedure to collect FF [30, 31]. For all experiments, FF was obtained from mature follicles (diameter ≥18 mm) and the protein samples were prepared as follows. FF samples were collected when the oocytes were aspirated under the guidance of transvaginal ultrasound. During oocyte retrieval, 2.5 ml of macroscopically clear FF lacking visible blood contamination was collected from all participants. After centrifugation at 4,000 × g for 10 min at 4°C, the supernatant liquid was drawn and immediately frozen in liquid nitrogen and stored at −80°C until use.

(1) Protein extraction: the FF samples were denatured using five volumes of cold acetone, precipitated at −20°C overnight, and centrifuged at 25,000  × g and 4°C for 15 min, and the supernatant was discarded. The precipitate was air-dried, sonicated three times in an ice bath (frequency 50 Hz), and centrifuged at 25,000  × g and 4°C for 15 min to obtain the supernatant. DTT was added to a final concentration of 10 mM and incubated at 56°C in a water bath for 1 h. IAM was added to a final concentration of 55 mM and incubated in the dark for 45 min, and the supernatant was obtained by centrifugation at 25,000  × g at 4°C for 15 min. The supernatant was the protein solution. (2) Protein quality control: the protein was quantified by the Bradford method [32]. (3) Protein enzymatic hydrolysis: a total of 100 *μ*g of protein solution per sample was diluted in four volumes of 50 mM NH_4_HCO_3_, and 2.5 *μ*g of trypsin enzyme was added (protein : enzyme = 40 : 1) and digested for 4 h at 37°C. The resulting peptides were desalted using a Strata X column and vacuumed to dryness. (4) Peptide fractionation: HPLC analysis was carried out on an LC-20AB liquid phase system (Shimadzu, Japan), and the separation column was a Gemini C-18 column (4.6 mm × 250 mm). The dried peptide sample was redissolved with mobile phase A (5% ACN, pH 9.8), injected, and eluted at a flow rate of 1 mL/min with mobile phase B (95% ACN, pH 9.8) for 10 min. The elution peak was monitored at a wavelength of 214 nm, and one component was collected per minute. All of the samples were combined according to the chromatographic elution peak map to obtain 10 fractions, which were then freeze-dried.

### 2.4. Data-Dependent Acquisition (DDA) and DIA Analysis by Nano-LC-MS/MS

The extracted peptide sample was redissolved with mobile phase A (2% ACN, 0.1% FA) and centrifuged at 20,000  × g for 10 min at 4°C, and the supernatant was taken for injection. The sample was first concentrated and desalted in a trap column and then connected in series with a self-assembled C18 column (35 cm × 150 *μ*m, 1.8 *μ*m). The proteins were separated on a Thermo UltiMate 3000 UHPLC liquid chromatograph at a flow rate of 500 nL/min with the following effective gradient: 0–5 min, 5% mobile phase B (98% ACN, 0.1% FA); 5–130 min, from 5% B to 25% B; 130–150 min, from 25% B to 35% B; 150–160 min, from 35% B to 80% B, 160–175 min, 80% B; and 175–180 min, from 80% B to 5% B. The peptides separated by liquid phase were ionized by a nano-ESI source and injected into a tandem mass spectrometer Fusion Lumos (Thermo Fisher Scientific, San Jose, CA) for DDA mode detection.

For DDA analysis, the main parameters were as follows: ion source voltage of 2 kV, MS scan range of 350–1,500 m/z, MS resolution of 120,000, maximum ion implantation time (MIT) of 50 ms, MS/MS collision type of HCD, collision energy NCE of 30, MS/MS resolution of 30,000, MIT of 100 ms, and dynamic exclusion of 30 s. The start m/z for MS/MS was fixed at 100. Precursors for the MS/MS scan satisfied the charge range of 2+ to 6+, and the top 20 precursors had intensities greater than 2E4. The AGC was MS 4E5, MS/MS 5E4.

For DIA analysis, LC-separated peptides were ionized by nano-ESI and injected into a Fusion Lumos tandem mass spectrometer (Thermo Fisher Scientific, San Jose, CA) in DIA mode. The main parameters were ion source voltage of 2 kV, MS scan range of 400–1500 m/z, MS resolution of 60,000, and MIT of 50 ms, and the 400–1500 m/z range was equally divided into 44 continuous window MS/MS scans. The MS/MS collision type was HCD, and MIT was 54 ms. Fragment ions were scanned in Orbitrap with an MS/MS resolution of 30,000, collision energy of 30, and AGC 5E4.

### 2.5. Data Analysis

Proteins in the DDA data were identified using MaxQuant (http://www.maxquant.org), and the identification results were used for spectral library construction [33]. For large-scale DIA data, the mProphet algorithm was used to perform the analytical quality control, thus obtaining a large number of reliable quantitative results. The identified proteins from spermatozoa were analyzed by gene ontology (GO) (http://david.abcc.ncifcrf.gov/home.jsp) and the Kyoto Encyclopedia of Genes and Genomes Database (KEGG) (http://www.genome.jp/kegg). Principal component analysis (PCA) of the quantified proteins was performed with the Unscrambler software (version 9.8). Based on the quantitative results, the differentially expressed proteins (DEPs) between the comparison groups were identified, and we performed functional enrichment analysis, protein-protein interaction analysis, and subcellular localization analysis of the DEPs using the Web tool STRING (http://string-db.org). Other databases for bioinformatic analysis were the UniProt protein database and the NCBI databases (including GenBank, RefSeq, Swiss-Prot, and PDB). The sex hormone data were analyzed by Student's *t*-test in SPSS 22.0, where *P* < 0.05 was considered significant.

## 3. Results

### 3.1. Efficacy Evaluation of KLP

The clinical features of the study participants are summarized in the supplementary data (Table S2). Age, body mass index, and serum levels of E_2_, FSH, LH, homocysteine, high-density lipoprotein cholesterol, low-density lipoprotein cholesterol, triglycerides, and lipoprotein were not statistically significantly different between the NOR and DOR groups (*P* > 0.05).

**Figure 3:**
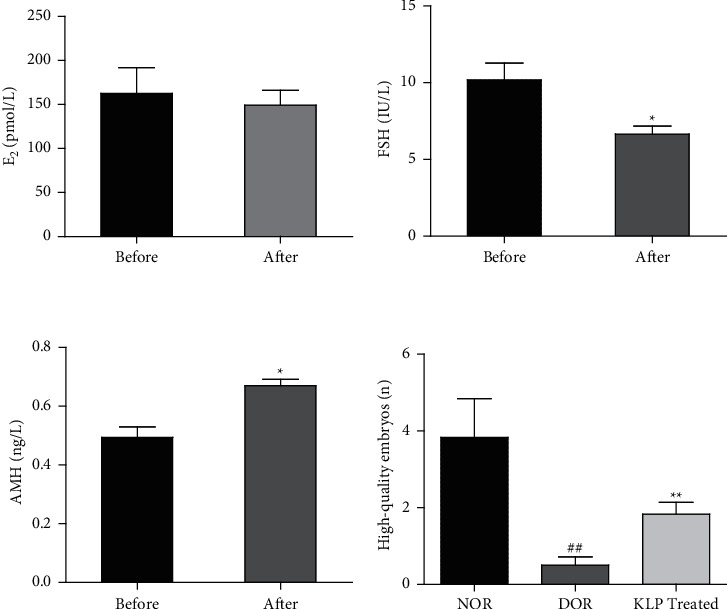
Serum levels of E_2_, FSH, and AMH and the number of high-quality embryos. The values are shown as the mean ± SEM (*n* = 6 per group). (a) After treatment with KLP, the levels of E_2_ were not significantly different compared with before treatment. (b) The level of FSH after treatment was significantly lower compared with before treatment (^*∗*^*P* < 0.05). (c) The level of AMH was significantly higher compared with before treatment (^*∗*^*P* < 0.05). (d) The number of high-quality embryos in the three groups. DOR group compared with the NOR group, (^##^*P* < 0.01); KLP treatment group compared with the DOR group (^*∗∗*^*P* < 0.01).

The levels of E_2_ were not significantly different before and after treatment with KLP (Figure 3(a)). However, compared with before treatment the levels of FSH decreased significantly after treatment with KLP (Figure 3(b)), while the levels of AMH were significantly elevated (Figure 3(c)). Moreover, compared with the NOR group the number of high-quality embryos in the DOR group decreased significantly, while the number of high-quality embryos increased significantly in the KLP treatment group compared with DOR groups (Figure 3(d)).

### 3.2. Quantitative Protein Detection

**Figure 4:**
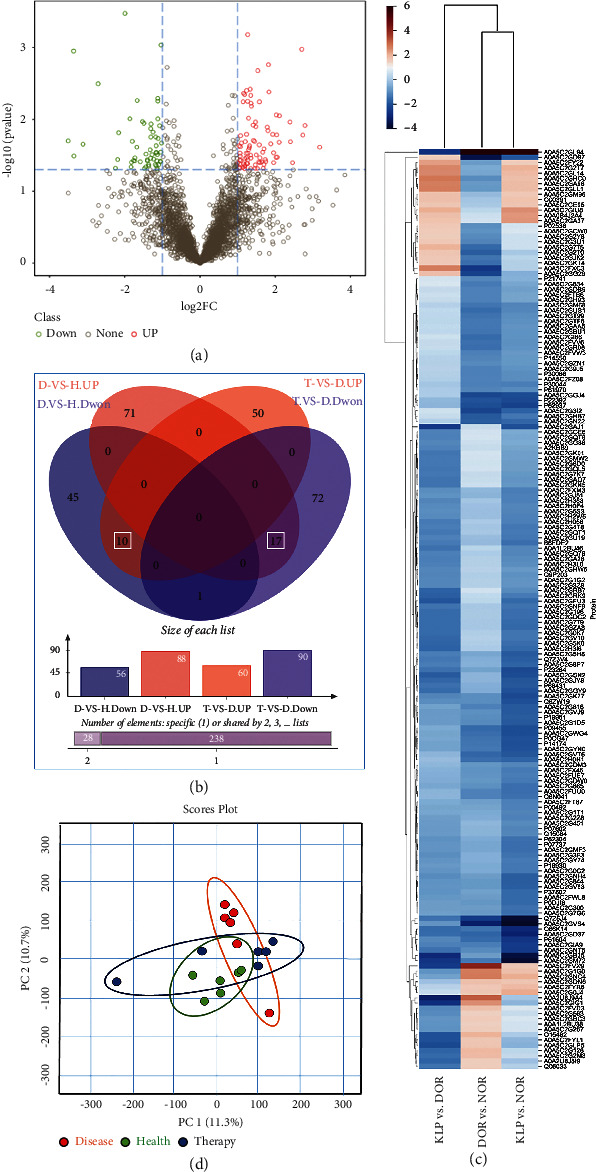
DDA and quantitative protein detection. (a) Identification of DEPs in DOR FF. (b) Venn diagram of the DEPs between the DOR group and the KLP-treated group. (c) Cluster analysis chart of the identified DEPs (higher red and blue intensities indicate a higher degree of upregulation and downregulation, respectively). (d) PCA chart.

Conventional DDA-MS was used to establish and analyze a spectral library of human FF obtained from 18 subjects, i.e., 6 NOR patients, 6 DOR patients, and 6 DOR patients treated with KLP. We identified 10,887 peptides and 3,774 proteins, and then, the DIA method was adopted for MS data collection. After calculating the fold changes and *P* value through the MSstats package, two filtration criteria (fold change >2 and *P* value <0.05) were used to identify significant DEPs. The differences between the comparison groups are presented in Figures 4(a) and 4(b). According to the Venn diagram, 56 proteins were downregulated and 88 proteins were upregulated in the DOR group compared with the NOR group, and 27 of the proteins were shared in the KLP-treated group. Among these, 10 proteins were upregulated and 17 proteins were downregulated in the KLP group compared with the DOR group. The identified proteins are listed and described in Table 1. In addition, all DEPs were used to draw a cluster analysis chart (Figure 4(c)), which intuitively illustrates the expression differences among the three groups. PCA of the qualified FF proteins showed that samples from NOR (health), DOR (disease), and KLP-treated (therapy) were in separate clusters (Figure 4(d)). The first two PCs explained 22% of the total variance and could distinguish the three species.

### 3.3. Bioinformatic Analyses

**Figure 5:**
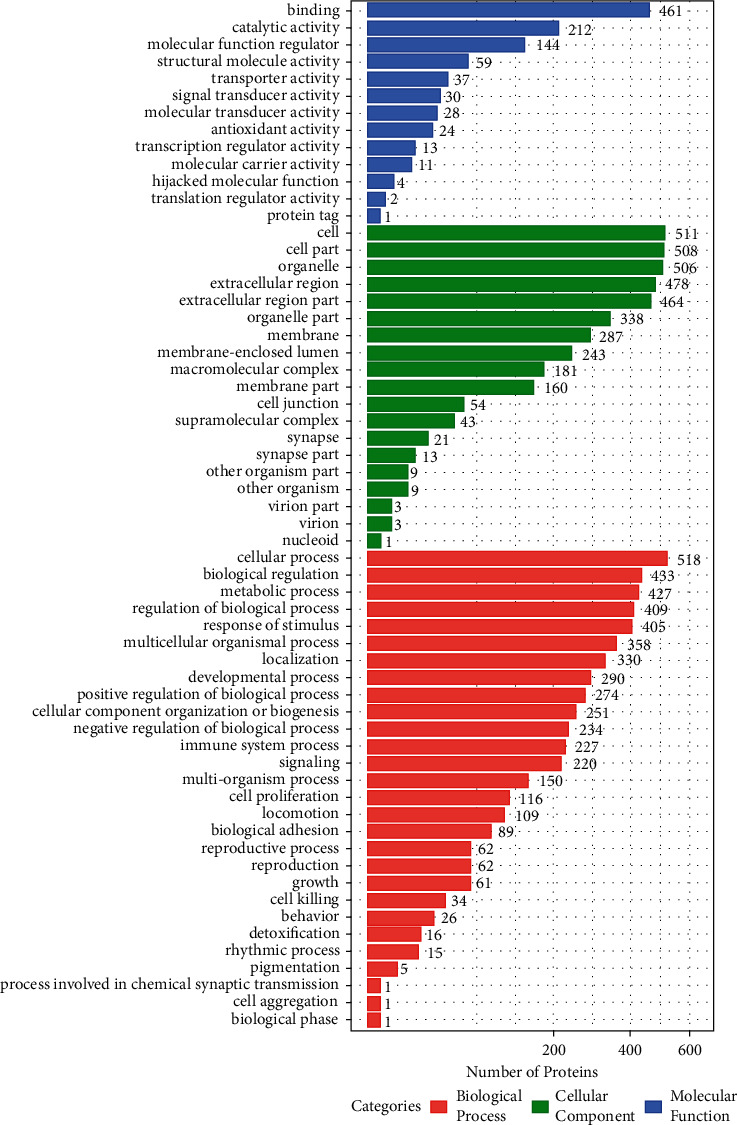
Bar graph of gene ontology (GO) classification of all identified FF proteins by DDA and quantitative protein detection. The length shows the number of DEPs associated with the GO term. The large numbers of proteins in the categories of metabolic process and biological process provide strong support for our hypothesis.

To evaluate the functional significance of all of the identified proteins, the Blast2GO software was used to perform gene ontology (GO) annotation analysis. As shown in Figure 5, the most enriched molecular functions accounted for 13 GO terms, such as binding, catalytic activity, and antioxidant activity. The most enriched cell components accounted for 19 GO terms, such as cell, cell part, and organelles. The most enriched biological processes accounted for 28 GO terms, such as cellular process, biological regulation, metabolic process, regulation of biological process, and response to stimuli.

**Figure 6:**
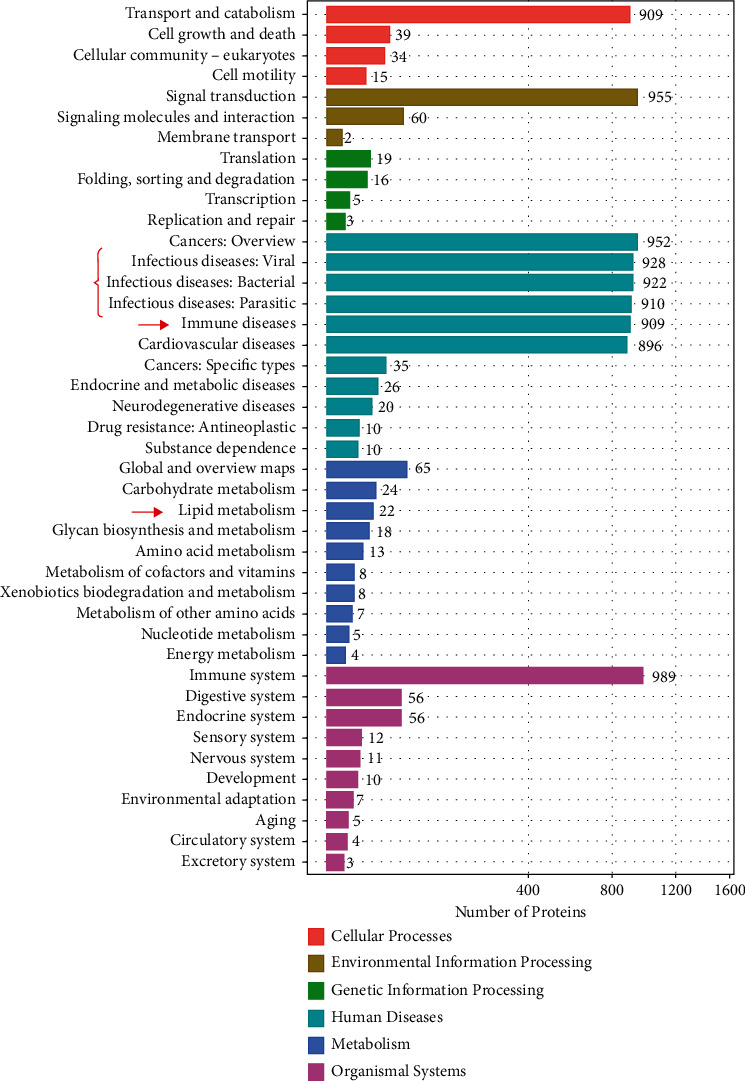
Bar graph of the KEGG pathway analysis of DEPs in the KLP-treated group vs. the DOR group. The bar length shows the number of all DEPs associated with the GO and KEGG terms.

According to the KEGG pathway enrichment analysis, the six highest ranked biological functions for the DEPs included cellular processes, environmental information processing, genetic information processing, human disease, metabolism, and organic systems (Figure 6). In the context of this experiment, the pathway enrichment analysis gave a snapshot of the significantly enriched metabolic pathways.

**Figure 7:**
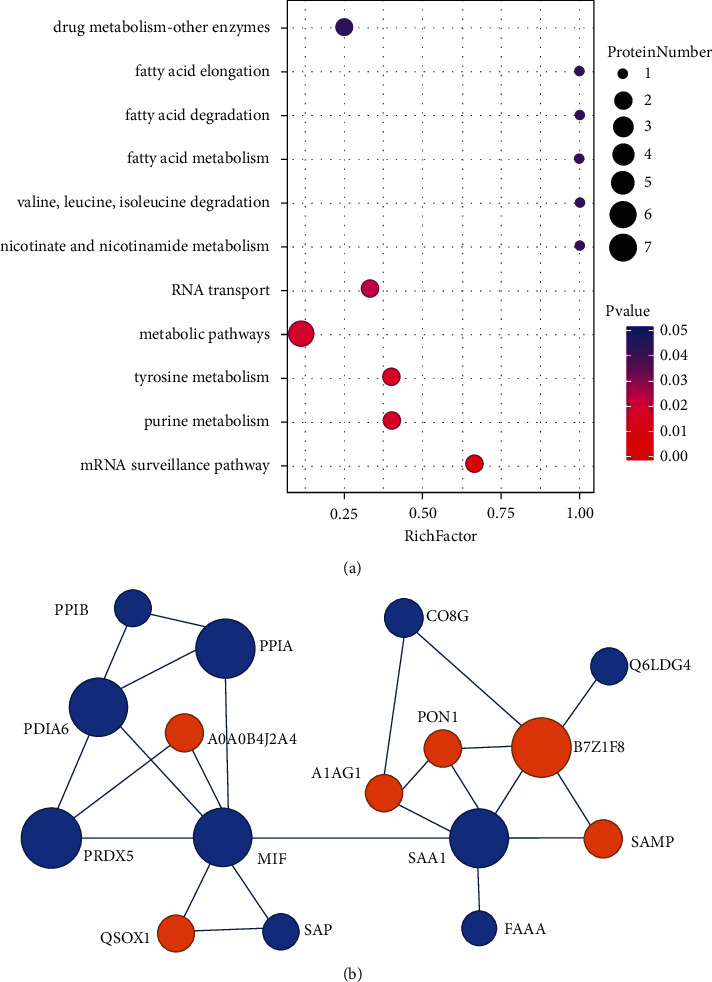
A bubble chart classification of the KEGG pathway analysis and the linking protein-protein interaction network between the KLP-treated group and the DOR group. (a) The bubble size shows the number of DEPs associated with each GO and KEGG term. (b) The red bubbles represent upregulated proteins, and the blue bubbles represent downregulated protein.

KEGG enrichment analysis of the DEPs identified in this study showed the involvement of multiple pathways, and infection, immune diseases, and lipid metabolism were considered to be endogenous signals affecting follicular development. Among these pathways, the 11 highest ranked biological functions for the DEPs in the KLP-treated group compared with the DOR group were mostly associated with fatty acid elongation, fatty acid degradation, fatty acid metabolism, nicotinate and nicotinamide metabolism, and valine, leucine, and isoleucine degradation (Figure 7(a)). The linking protein information is listed in Table 2. We then imported the protein IDs into the STRING database to build a protein-protein network (Figure 7(b)). Three proteins (MIF, SAA1, and PRDX5) were core nodes and were connected to two subnetworks, one involving SAA1, FAAA, SAMP, A1AG1, PON1, B7Z1F8, CO8G, and Q6LDG4 and the other involving MIF, SAP, QSOX1, PRDX5, PPIA, PDIA6, A0A0B4J2A4, and PPIB.

## 4. Discussion

Infertility has become a worldwide public health problem, affecting about 12.5–15 in 100 couples of reproductive age annually in China [34]. DOR, generally defined as a decreased number of high-quality oocytes, is a predominant contributor to infertility [35–37]. In recent years, the incidence of DOR has been increasing and the trend is for it to present in younger patients. Thus, the development of effective treatment strategies has emerged as one of the preeminent topics in the reproductive health field.

KLP, also called Kunling Wan, has been demonstrated to increase endometrial blood flow, upregulate vascular endothelial growth factor A, and inhibit angiogenesis and endometriosis induced by controlled ovarian hyperstimulation [38]. Some published clinical studies have suggested that KLP has a better therapeutic effect on DOR and premature ovary failure (ref. in Chinese). Our experimental results partly support the existing arguments, but the pharmacological mechanisms of KLP in the treatment of DOR remain unclear.

A major strength of this study is that we used human FF collected from IVF patients with or without DOR and with DOR treated by KLP. The proteomic profiles of the FF samples were obtained by DIA-MS combined with bioinformatic analyses. The quantitative analysis results suggested that 27 proteins (10 upregulated and 17 downregulated) were differentially expressed between the NOR patients, DOR patients, and KLP-treated patients. Among these, 4 proteins were associated with protein degradation, 4 proteins were involved in the immune response, and 16 proteins were associated with various biological functions such as inflammatory response, free radical scavenging, protein modification, and energy conversion. It is expected therefore that these proteins have essential roles in DOR patients treated by KLP. Regarding these DEP functions, the results of GO and KEGG enrichment analyses indicated that most of the annotations belonged to binding, catalytic activity, cellular process, biological regulation, and metabolic process. Our datasets are in agreement with previous reports in similar projects [39, 40].

In our study, some signature proteins in the FF were mainly found after KLP treatment. Among them, macrophage migration inhibitory factor (MIF) is a soluble pro-inflammatory cytokine produced by activated T lymphocytes that triggers cell proliferation, migration, follicle growth, and ovulation [41]. MIF is also an important regulator of the host innate immunity induced by pro-inflammatory states such as PCOS and ovarian tumors [42, 43], and it has been associated with various immunological events in the process of oocyte development [44, 45]. SAA1 (serum amyloid A), an acute-phase protein, is produced mainly by the liver and ovarian granulosa cells [46]. The biological functions of SAA1 in the ovary are still not fully understood, but a previous study demonstrated that elevated follicular SAA1 is associated with decreased pregnancy rate [47], and our findings suggest that SAA1 is a potential target of KLP in the treatment of DOR. PRDXs (peroxiredoxins) are cytoprotective peroxidases that prevent oxidative stress by reducing peroxides. PRDXs constitute a large superfamily (PRDX1–6) of proteins that are involved in the processes of inflammation and tumor development, including ovarian cancer [48]. In particular, PRDX5 plays an important role in the Nrf2 signaling pathway [49]. Linking with the three core proteins, multiple proteins such as alpha-1-acid glycoprotein-1 (A1AG1), paraoxonase-1 (PON1), acyl-CoA synthetase gene (FAAA), serum amyloid P component (SAP), quiescin sulfhydryl oxidase-1 (QSOX1), protein disulfide-isomerase family-6 (PDIA6), and cyclophilins (PPIA and PPIB) co-regulate lipid acid metabolism, nicotinate and nicotinamide metabolism, and tyrosine and purine metabolism.

## 5. Conclusions

The FF is different from blood and is a unique biological fluid in which the critical events of oocyte and follicular maturation take place, and it provides a unique window into the processes occurring during follicular maturation. In summary, our study provides the proteome profiles of human FF from DOR patients with and without KLP treatment, and functional analyses of proteome datasets revealed a possible pharmacological mechanism of KLP for the improvement of DOR. Several detected core proteins (SAA1, MIF, PRDX5) and related pathways (fatty acid metabolism, nicotinate and nicotinamide metabolism, and tyrosine and purine metabolism) are potential targets of KLP. Our proposed datasets provide a useful basis for future studies to better understand the pathological mechanisms of DOR and the pharmacological mechanisms of TCM preparations.

## 6. Limitations

Although DIA-MS in this study can provide the possibility to investigate proteomic changes in the FF, the conventional bioinformatic analysis methods might miss some possible valuable information. Adding the effect of the smaller sample size, caution should be taken in extrapolating this result to other studies and to clinical practice. In addition, experimental verification was not carried out in this study, and we are now designing a cross-validation using other omic methods such as transcriptomics or metabolomics.

## Figures and Tables

**Table 1:** Differentially expressed proteins in the KLP-treated group compared with the DOR group.

Swiss-Prot Id	Gene name	Description	Up or down
O00391	QSOX1	Sulfhydryl oxidase 1	↑
P27169	PON1	Serum paraoxonase/arylesterase 1	↑
A0A0B4J2A4	ACAA2	3-Ketoacyl-CoA thiolase, mitochondrial	↑
P02763	A1AG1	Alpha-1-acid glycoprotein 1	↑
B7Z1F8	B7Z1F8	cDNA FLJ53025	↑
Q5SNT2	SAMP	Transmembrane protein 201	↑
P07360	CO8G	Complement component C8 gamma chain	↑
Q5T4S7	UBR4	E3 ubiquitin-protein ligase UBR4	↑
Q6LDG4	C2	Complement protein	↑
A0A5C2H1W2	N	IG c1918_light_IGLV1-51_IGLJ3	↑
P16930	FAH	Fumarylacetoacetase	↓
P0DJI8	SAA1	Serum amyloid A-1 protein	↓
P14174	MIF	Macrophage migration inhibitory factor	↓
O75446	SAP	Histone deacetylase complex subunit SAP30	↓
P30044	PRDX5	Peroxiredoxin-5	↓
P23284	PPIB	Peptidyl-prolyl cis-trans isomerase B	↓
P62937	PPIA	Peptidyl-prolyl cis-trans isomerase A	↓
Q15084	PDIA6	Protein disulfide-isomerase A6	↓
P07602	PSAP	Prosaposin	↓
P62304	SNRPE	Small nuclear ribonucleoprotein E	↓
P68431	H3C1	Histone H3.1	↓
Q15084	PDIA6	Protein disulfide-isomerase A6	↓
Q7Z3V4	UBE3B	Ubiquitin-protein ligase E3B	↓
Q7Z5J4	RAI1	Retinoic acid-induced protein 1	↓
A0A0B4J2A4	ACAA2	3-Ketoacyl-CoA thiolase, mitochondrial	↓
B6EDE2	HEL180	Epididymis luminal protein 180	↓
P00492	HPRT1	Hypoxanthine-guanine phosphoribosyltransferase	↓

**Table 2:** Proteins contained in the significantly enriched pathways.

Pathway ID	Pathway	Matched proteins	Functional description
map00062	Fatty acid elongation	A0A0B4J2A4, SAA1	Lipid metabolism
map00071	Fatty acid degradation		
map01212	Fatty acid metabolism		
map00280	Valine, leucine, and isoleucine degradation		
map00760	Nicotinate and nicotinamide metabolism	RET1	Metabolism of cofactors and vitamins
map03015	mRNA surveillance pathway	NTF2, Q6ZW19 NTF2, Q6ZW19	Genetic information processing, translation
map03013	RNA transport		
map00350	Tyrosine metabolism	MIF, FAAA	Amino acid metabolism
map00230	Purine metabolism	HPRT, NDKB	Nucleotide metabolism

## Data Availability

All datasets used to support the findings of the study are included within the article and supplementary files.

## Abbreviations

DOR: Diminished ovarian reserve
TCM: Traditional Chinese medicine
ART: Assisted reproductive technology
FF: Follicular fluid
DDA: Data-dependent acquisition
DIA: Data-independent acquisition
DEP: Differentially expressed proteins
GO: Gene ontology
KEGG: Kyoto Encyclopedia of Genes and Genomes Database
PCOS: Polycystic ovary syndrome
MS: Mass spectrometry
IVF: In vitro fertilization
FSH: Follicle-stimulating hormone
E_2_: Estradiol
AMH: Anti-Müllerian hormone
PCA: Principal component analysis
LH: Luteinizing hormone.

## Supplementary Materials

Supplementary Materials Table S1: components of KLP. Table S2: the baseline clinical parameters of the study participants before KLP treatment. Table S3: the main effective ingredients of KLP.
