# Supplementary material for: Proteomic Analysis of Human Follicular Fluid Reveals the Pharmacological Mechanisms of the Chinese Patent Drug Kunling Pill for Improving Diminished Ovarian Reserve
Source: Evid Based Complement Alternat Med. 2022 May 28;2022:5929694. doi: 10.1155/2022/5929694 (PMC9167067; doi:10.1155/2022/5929694)
Supplement: Supplementary Materials — Table S1: components of KLP. Table S2: the baseline clinical parameters of the study participants before KLP treatment. Table S3: the main effective ingredients of KLP. [file 5929694.f1.zip › 5929694.f1/supplementary data table S1.docx]

Supplementary materials

S1: Table 1. Composition of KLP

|  | Species | Chinese name | Family | Ratio in formula  (%) |
| --- | --- | --- | --- | --- |
| 1 | Cyperus rotundus L | Xiangfu | Cyperaceae | 15.7 |
| 2 | Radix glycyrrhizae | Gancao | Papilionaceae | 1.5 |
| 3 | Radix cynanchi atrati | Baiwei | Asclepiadaceae | 3.0 |
| 4 | Herba leonuri | Yimucao | Lamiaceae | 3.0 |
| 5 | Radix scutellariae | Huangqi | Leguminosae | 3.0 |
| 6 | Celosia cristata | Jiguanhua | Amaranthaceae | 3.0 |
| 7 | Radix ophiopogonis | Maidong | Liliaceae | 3.0 |
| 8 | Fructus Schisandrae chinensis | Wuweizi | Magnoliaceae | 3.0 |
| 9 | Radix rehmanniae | Dihuang | Scrophulariaceae | 3.0 |
| 10 | Carthamus tinctorius L | Honghua | Asteraceae | 3.0 |
| 11 | Akebia quinata | Mutong | Lardizabalaceae | 2.1 |
| 12 | Atractylodes macrocephala | Baizhu | Asteraceae | 3.0 |
| 13 | Halloysitum rubrum | Chishizhi | Silicate | 3.0 |
| 14 | Poria cocos | Fuling | Polyporaceae | 3.0 |
| 15 | Magnolia officinalis | Houpu | Magnoliaceae | 2.1 |
| 16 | Cistanche salsa | Roucongrong | Orobanchaceae | 3.0 |
| 17 | Cynanchum otophyllum | Baishao | Paeoniaceae | 3.0 |
| 18 | Nepeta cataria L | Jingjie | Labiatae | 2.1 |
| 19 | Cortex moutan | Mudanpi | Ranunculaceae | 3.0 |
| 20 | Colla Corii Asini | Ejiao | Equidae | 3.0 |
| 21 | Angelica sinensis | Danggui | Lamiaceae | 3.0 |
| 22 | Ligusticum sinense Oliv | Gaoben | Umbelliferae | 2.1 |
| 23 | Panax ginseng C.A.Mey | Hongshen | Araliaceae | 3.0 |
| 24 | Deerhorn Glue | Lujiaojiao | Cervidae | 3.0 |
| 25 | Fritillaria cirrhosa D. Don | Chuanbeimu | Liliaceae | 3.0 |
| 26 | Nacre Concha Margaritifera Usta | Moyao | Burseraceae | 3.0 |
| 27 | Amomum villosum Lour | Sharen | Zingiberaceae | 3.0 |
| 28 | Corydalis | Yanhusuo | Papaveraceae | 3.0 |
| 29 | Foeniculum vulgare | Xiaohuixiang | Umbelliferae | 3.0 |
| 30 | Colla Carapacis et Plastri Testudinis | Guijiajiao | Testudinidae | 3.0 |
| 31 | Ligusticum wallichii | Chuanxiong | Apiaceae Lindl | 3.0 |
