# Supplementary material for: Proteomic Analysis of Human Follicular Fluid Reveals the Pharmacological Mechanisms of the Chinese Patent Drug Kunling Pill for Improving Diminished Ovarian Reserve
Source: Evid Based Complement Alternat Med. 2022 May 28;2022:5929694. doi: 10.1155/2022/5929694 (PMC9167067; doi:10.1155/2022/5929694)
Supplement: Supplementary Materials — Table S1: components of KLP. Table S2: the baseline clinical parameters of the study participants before KLP treatment. Table S3: the main effective ingredients of KLP. [file 5929694.f1.zip › 5929694.f1/supplementary data table S2.docx]

Supplementary materials

Table S2 The baseline clinical parameters of study participants before *KLP* treatment (mean ± standard deviation, n = 6)

| Feature | NOR | DOR | | F/x^2^ | *P* |
| --- | --- | --- | --- | --- | --- |
|  |  | No treatment | Before *KLP* treatment |  |  |
| age | 32.5 ± 1.76 | 37.16 ± 3.54 | 34.83 ± 6.27 | 1.78 | 0.21 |
| BMI | 28.28 ± 14.32 | 21.7 ± 3.58 | 23.6 ± 3.23 | 0.9 | 0.43 |
| Years of infertility (years) | 3.5 ± 1.87 | 3.75 ± 2.19 | 2.83 ± 3.54 | 0.19 | 0.83 |
| AFC(n) | 10.83 ± 2.64* | 4.1 ± 0.75^#^ | 2.67 ± 1.21^#^ | 26.24 | 0.01 |
| AMH (ng/L) | 3.65 ± 1.68* | 0.89 ± 0.14^#^ | 0.555 ± 0.14^#^ | 18.29 | 0.01 |
| Basal E_2_ levels (pg/ml) | 35.95 ± 12.04 | 42.5 ± 17.88 | 44.32 ± 19.47 | 0.41 | 0.67 |
| Basal FSH levels (IU/L) | 5.95 ± 0.57 | 10.8 ± 5.58 | 8.16 ± 3.43 | 2.45 | 0.12 |
| Basal LH levels (IU/L) | 5.04 ± 3.47 | 5.75 ± 2.31 | 2.91 ± 0.85 | 2.19 | 0.15 |
| Serum Hcy level (μmol/L) | 7.1 ± 1.69 | 8.28 ± 1.63 | 7.47 ± 1.55 | 0.84 | 0.45 |
| CHO (mmol/L) | 5.50 ± 1.35 | 4.39 ± 0.96 | 4.67 ± 0.17 | 2.15 | 0.15 |
| HDL-C (mmol/L) | 1.36 ± 0.19 | 1.38 ± 0.39 | 1.34 ± 0.25 | 0.02 | 0.98 |
| LDL-C (mmol/L) | 3.44 ± 1.17 | 2.55 ± 0.99 | 2.68 ± 0.14 | 1.76 | 0.21 |
| TG (mmol/L) | 1.97 ± 1.06 | 1.41 ± 0.83 | 2.11 ± 1.45 | 0.63 | 0.55 |
| Lpa (mg/L) | 232.33 ± 361.28 | 180.6 ± 153.66 | 170.83 ± 141.34 | 0.11 | 0.9 |

Note: Compared with the normal control group, ^#^*P* < 0.05; compared with the DOR control group, **P* < 0.05;

AFC: antral follicle count; AMH: anti-Müllerian hormone; E_2_: estrogen; FSH: follicle stimulating hormone; LH: luteinizing hormone; Hcy: homocysteine; CHO: cholesterol; HDL-C: high density lipoprotein-cholesterol C; LDL-C: low density lipoprotein-cholesterol C; TG: triglyceride; Lpa: lipoprotein.
