# Supplementary material for: Proteomic Analysis of Human Follicular Fluid Reveals the Pharmacological Mechanisms of the Chinese Patent Drug Kunling Pill for Improving Diminished Ovarian Reserve
Source: Evid Based Complement Alternat Med. 2022 May 28;2022:5929694. doi: 10.1155/2022/5929694 (PMC9167067; doi:10.1155/2022/5929694)
Supplement: Supplementary Materials — Table S1: components of KLP. Table S2: the baseline clinical parameters of the study participants before KLP treatment. Table S3: the main effective ingredients of KLP. [file 5929694.f1.zip › 5929694.f1/supplementary data table S3 (1).docx]

**S3: The main effective ingredients of Kuling pill**

1. Sample preparation

The coating was removed from a 3 g pill and it was ground into a powder. The powder was redissolved in 25 ml of methanol and ultrasonicated (power 250 W, frequency 50 kHz) for 40 min. The lost mass was made up with methanol, the solution was filtered, and the supernatant was subjected to HPLC analysis.

1. Content of the main components in Kunling pill

HPLC chromatogram of Kunling pill was shown in Figure 1.

2

1

Figure 1. HPLC chromatogram of KLP. (1: paeoniflorin; 2: paeonol)

Table 1. Content of the main components in *Kunling Pill*

| Lot No. | 1-Paeoniflorin (mg/pill) | 2-Paeonol (mg/pill) |
| --- | --- | --- |
| 20191022 | 0.3 | 0.085 |
